# Supplementary material for: COVID-19 hospitalization and mortality and hospitalization-related utilization and expenditure: Analysis of a South African private health insured population
Source: PLoS One. 2022 May 5;17(5):e0268025. doi: 10.1371/journal.pone.0268025 (PMC9070881; doi:10.1371/journal.pone.0268025)
Supplement: S1 Table — (DOCX) [file pone.0268025.s001.docx]

| S1 Table: Comparison of Discovery Health Administration profile versus rest of the rest of the insured population in South Africa | | | |
| --- | --- | --- | --- |
|  | **Study Population (Discovery Health membership)** | **Rest of the insured population (Membership of other medical schemes)** | **Total Insured Population** |
|  |  |  |  |
| Number of Families | 1,659,247 | 3,110,948 | 4,770,195 |
| Number of lives covered | 3,480,943 | 7,311,044 | 10,791,987 |
| No. of dependents per family | 2.0979 | 2.3501 | 2.2624 |
| Average contributions (in SA Rands)* | 2,039 | 1,863 | 1,920 |
| Average Health care expenditure* | 1,500 | 1,613 | 1,577 |

* Per life per annum
