# Supplementary material for: COVID-19 hospitalization and mortality and hospitalization-related utilization and expenditure: Analysis of a South African private health insured population
Source: PLoS One. 2022 May 5;17(5):e0268025. doi: 10.1371/journal.pone.0268025 (PMC9070881; doi:10.1371/journal.pone.0268025)
Supplement: S2 Table — (DOCX) [file pone.0268025.s002.docx]

S2 Table: Univariate and multivariate analysis of factors associated with hospitalisation cost with coefficients

| Cost per hospitalised COVID-19 patient (SA Rands) | | | | |
| --- | --- | --- | --- | --- |
| Variable | Total hospitalised COVID-19 cases | Median cost/ patient (IQR) | Unadjusted coefficient (95%CI) | Adjusted coefficient (95%CI) |
| Age |  |  |  |  |
| Less than 18 | 1,204 | 24,400 (15,552 – 39,586) | Reference | Reference |
| Between 18-25 | 621 | 30,390 (18,944 – 54,741) | 0.19 (0.09, 0.29) | 0.24 (0.13, 0.34) |
| Between 25-40 | 7,357 | 42,051 (24,974 – 68,435) | 0.48 (0.42, 0.55) | 0.47 (0.41, 0.54) |
| Between 40-65 | 17,384 | 57,801 (33,276 – 138,005) | 0.95 (0.89, 1.01) | 0.84 (0.78, 0.90) |
| Greater than 65 | 8,901 | 83,335 (41,290 – 203,098) | 1.15 (1.09, 1.21) | 1.00 (0.93, 1.07) |
| Gender |  |  |  |  |
| Female | 17,431 | 50,162 (28,650 – 103,434) | Reference | Reference |
| Male | 18,036 | 60,793 (33,151 – 155,153) | 0.24 (0.21, 0.26) | 0.17 (0.15, 0.19) |
| Province |  |  |  |  |
| Western Cape | 7,248 | 47,080 (26,290 – 103,145) | Reference | Reference |
| Eastern Cape | 1,902 | 48,843 (28,421 – 101,642) | 0.01 (-0.04, 0.07) | -0.01 (-0.06, 0.05) |
| Free State | 1,078 | 45,547 (27,073 – 84,580) | -0.06 (-0.13, 0.01) | -0.02 (-0.08, 0.05) |
| Gauteng | 13,514 | 54,059 (30,663 – 117,780) | 0.14 (0.11, 0.17) | 0.11 (0.08, 0.14) |
| KwaZulu-Natal | 7,705 | 50,388 (29,753 – 107,514) | 0.09 (0.05, 0.12) | 0.04 (0.01, 0.08) |
| Limpopo | 717 | 36,514 (21,760 – 73,986) | -0.23 (-0.31, -0.14) | -0.16 (-0.24, -0.08) |
| Mpumalanga | 1,279 | 41,264 (23,406 – 91,366) | -0.11 (-0.18, -0.05) | -0.04 (-0.11,0.02) |
| North West | 1,316 | 47,634 (27,784 – 88,583) | -0.01 (-0.08, 0.05) | -0.00 (-0.06, 0.06) |
| Northern Cape | 599 | 39,213 (23,116 – 69,723) | -0.22 (-0.31, -0.13) | -0.11 (-0.19, -0.02) |
| # of comorbidities |  |  |  |  |
| 0 | 15,110 | 42,079 (24,547 – 76,712) | Reference | Reference |
| 1 | 6,758 | 50,359 (29,152 – 107,048) | 0.22 (0.19, 0.25) | 0.07 (0.04, 0.11) |
| 2 | 5,744 | 58,489 (33,018 – 132,798) | 0.38 (0.34, 0.41) | 0.16 (0.13, 0.19) |
| 3 | 4,243 | 62,213 (34,300 – 148,067) | 0.44 (0.41, 0.48) | 0.18 (0.15, 0.22) |
| >3 | 3,612 | 73,423 (37,685 – 170,703) | 0.55 (0.51, 0.59) | 0.25 (0.21, 0.29) |
| Pandemic wave |  |  |  |  |
| Pre-Wave 1 | 1,091 | 52,011 (27,318 – 120,072) | 0.06 (-0.00, 0.13) | 0.13 (0.06, 0.19) |
| Wave 1 | 11,884 | 48,577 (27,537 – 103,472) | Reference | Reference |
| Post-wave 1 | 3,662 | 45,169 (25,009 – 90,903) | -0.11 (-0.15, -0.07) | -0.03 (-0.07, 0.01) |
| Wave 2 | 18,380 | 51,524 (29,882 – 112,648) | 0.07 (0.05, 0.10) | 0.07 (0.04, 0.09) |
| Medical insurance cover level |  |  |  |  |
| Level 1 | 4,796 | 44,071 (24,695 – 91,445) | Reference | Reference |
| Level 2 | 4,554 | 50,698 (29,034 – 114,502) | 0.19 (0.14, 0.23) | 0.08 (0.04, 0.12) |
| Level 3 | 21,088 | 49,326 (28,477 – 102,979) | 0.15 (0.12, 0.18) | 0.08 (0.05, 0.12) |
| Level 4 | 4,833 | 59,609 (32,486 – 131,414) | 0.32 (0.28, 0.36) | 0.08 (0.04, 0.12) |
| Private Hospital network |  |  |  |  |
| Network A | 1,307 | 53,407 (30,403 – 123,034) | Reference | Reference |
| Network B | 9,601 | 59,086 (35,733 – 128,490) | 0.11 (0.05, 0.17) | 0.05 (-0.01, 0.11) |
| Network C | 546 | 36,068 (22,360 – 69,732) | -0.44 (-0.55, -0.33) | -0.32 (-0.42, -0.21) |
| Network D | 5,407 | 49,165 (27,202 – 101,265) | -0.13 (-0.19, -0.06) | -0.13 (-0.19, -0.07) |
| Network E | 9,151 | 44,706 (25,529- 91,528) | -0.19 (-0.25, -0.13) | -0.19 (-0.25, -0.13) |
| Network F | 8,846 | 47,670 (27,554 – 105,322) | -0.11 (-0.17, -0.04) | -0.13 (-0.19, -0.07) |
